# Seasonal Ely Copper Mine Superfund site shotgun metagenomic and metatranscriptomic data analysis

**DOI:** 10.1016/j.dib.2020.106282

**Published:** 2020-09-08

**Authors:** Lesley-Ann Giddings, George Chlipala, Heather Driscoll, Kieran Bhave, Kevin Kunstman, Stefan Green, Katherine Morillo, Holly Peterson, Mark Maienschein-Cline

**Affiliations:** a Department of Chemistry & Biochemistry, Middlebury College, Middlebury, VT 05753, USA; b Department of Chemistry, Smith College, Northampton, MA 01063, USA; c Vermont Genetics Network, Department of Biology, Norwich University, Northfield, VT 05663, USA; d Research Resources Center, University of Illinois at Chicago, Chicago, IL 60612, USA; e Department of Geology, Guilford College, Greensboro, NC 27403, USA

**Keywords:** Acid rock drainage, Metagenome, Metatranscriptome, Illumina NextSeq, Differential analysis

## Abstract

High throughput sequencing data collected from acid rock drainage (ARD) communities can reveal the active taxonomic and functional diversity of these extreme environments, which can be exploited for bioremediation, pharmaceutical, and industrial applications. Here, we report a seasonal comparison of a microbiome and transcriptome in Ely Brook (EB-90M), a confluence of clean water and upstream tributaries that drains the Ely Copper Mine Superfund site in Vershire, VT, USA. Nucleic acids were extracted from EB-90M water and sediment followed by shotgun sequencing using the Illumina NextSeq platform. Approximately 575,933 contigs with a total length of 1.54 Gbp were generated. Contigs of at least a size of 3264 (N50) or greater represented 50% of the sequences and the longest contig was 488,568 bp in length. Using Centrifuge against the NCBI “nt” database 141 phyla, including candidate phyla, were detected. Roughly 380,000 contigs were assembled and ∼1,000,000 DNA and ∼550,000 cDNA sequences were identified and functionally annotated using the Prokka pipeline. Most expressed KEGG-annotated microbial genes were involved in amino acid metabolism and several KEGG pathways were differentially expressed between seasons. Biosynthetic gene clusters involved in secondary metabolism as well as metal- and antibiotic-resistance genes were annotated, some of which were differentially expressed, colocalized, and coexpressed. These data can be used to show how ecological stimuli, such as seasonal variations and metal concentrations, affect the ARD microbiome and select taxa to produce novel natural products. The data reported herein is supporting information for the research article “Characterization of an acid rock drainage microbiome and transcriptome at the Ely Copper Mine Superfund site” by Giddings et al. [1].

## Specifications Table

**Table:** 

Subject	Microbial Ecology, Genomics and Molecular Biology
Specific subject area	Metagenomics
Type of data	Tables, figures, raw data
How data were acquired	Shotgun metagenomic and metatranscriptomic sequence data were acquired using an Illumina NextSeq500 instrument. Centrifuge was used to perform a read-based taxonomic analysis of metagenomic data. Prokka was used to detect and functionally annotated open reading frames. The predicted amino acid sequence was searched against Swiss-Prot database using DIAMOND. KEGG orthology annotations were predicted for open reading frames. All differential and statistical analyses on taxonomic summaries were performed in edgeR [2]. BacMet [3], antiSMASH 5.0 [4], ARTS version 2.0 [5] databases were used to annotate genes.
Data format	Annotated data, Bray-Curtis dissimilarity matrices, Non-metric multidimensional scaling (NMDS) plots, principal component analysis (PCA) plots, heat map and hierarchal clustering, raw count data, and gradient plots.
Parameters for data collection	Seasonal environmental water and sediment samples were collected and sequenced. Five water and three sediment samples from summer as well as three sediment samples from winter.
Description of data collection	Shotgun metagenomic and metatranscriptomic sequencing was performed using an Illumina NextSeq500 instrument.
Data source location	Sediment (July 28th, 2017 and January 14th, 2018) and water (July 14th, 2017 and July 28th, 2017) samples were collected 90 m upstream from the mouth of Ely Brook (EB-90M) at Ely Copper Mine, Vershire, VT, USA (43°55′9″ N, 72°17′11″ W).
Data accessibility	Data are shown in this article. Raw metagenomic and metatranscriptomic data have been deposited in the Sequence Read Archive of the National Center for Biotechnology Information (BioProject identifier, PRJNA540505). Taxonomic and functional annotations as well as normalized count data used for all analyses are available in a public repository: Repository name: FigShare Data identification number: 10.6084/m9.figshare.c.4864863 Direct URL to data: https://doi.org/10.6084/m9.figshare.c.4864863
Related research article	L.-A. Giddings, G. Chlipala, K. Kunstman, S. Greene, K. Morillo, K. Bhave, H. Peterson, H. Driscoll, M. Maienschein-Cline, Characterization of an acid rock drainage microbiome and transcriptome at Ely Copper Mine Superfund site, PLoS One, 15(8) (2020) e0237599. https://doi.org/10.1371/journal.pone.0237599

## Value of the Data

• This is the first characterization of an acid rock drainage (ARD) metagenome and transcriptome within the Vermont copper belt region, USA, which is comprised of Ely Copper Mine, Elizabeth Mine, and Pike Hill Copper Mine.
• The metagenomic data provide seasonal taxonomic profiles of the microbial diversity in the sediment and water of EB-90M.
• Active taxa in ARD environments are understudied and the metagenomic and metatranscriptomic data provide insight into their seasonal functional roles within these acidic, metal-rich environments.
• These data can be used to perform comparative taxonomic and functional analyses with other ARD metagenomes.
• These data can be used to bioprospect enzymes that can be exploited for the bioremediation of metal polluted environments.
• These data can be used to identify novel genes encoding proteins involved in the production of bioactive secondary metabolites, which can be used for pharmaceutical and industrial applications.

## 2 Data Description

**Fig. 1:**
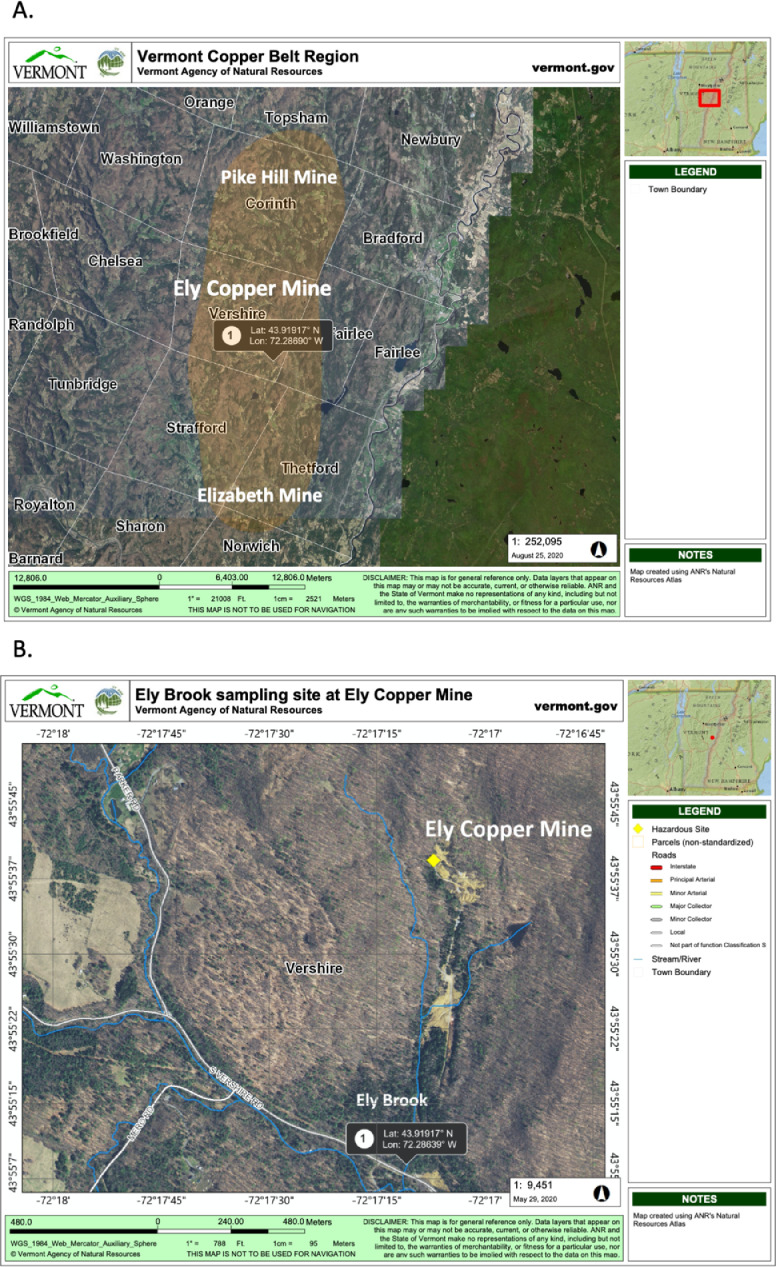
*Vermont copper belt.* A) Map of Vermont copper belt (highlighted in yellow), which includes Ely Copper Mine (sampling site), Pike Hill Mine, and Elizabeth Mine. B) Map of Ely Brook sample site, which drains Ely Copper Mine. (For interpretation of the references to color in this figure legend, the reader is referred to the web version of this article.)

**Fig. 2:**
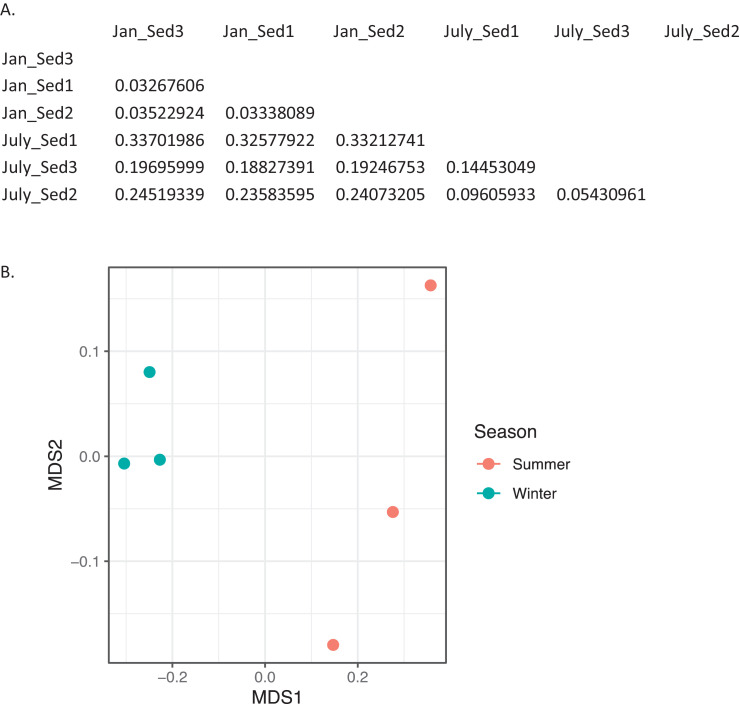
*Bray-Curtis dissimilarity indices for archaea in sediment.* A) Matrix of dissimilarity indices calculated for genera of archaea in sediment samples using the Bray-Curtis method. 'Sed' = sediment. B) NMDS plot to visualize the dissimilarity between genera of archaea in summer (July_Sed1, July_Sed2, and July_Sed3 in orange) and winter (Jan_Sed1, Jan_Sed2, and Jan_Sed3 in blue) sediment collected at EB-90M. (For interpretation of the references to color in this figure legend, the reader is referred to the web version of this article.)

**Fig. 3:**
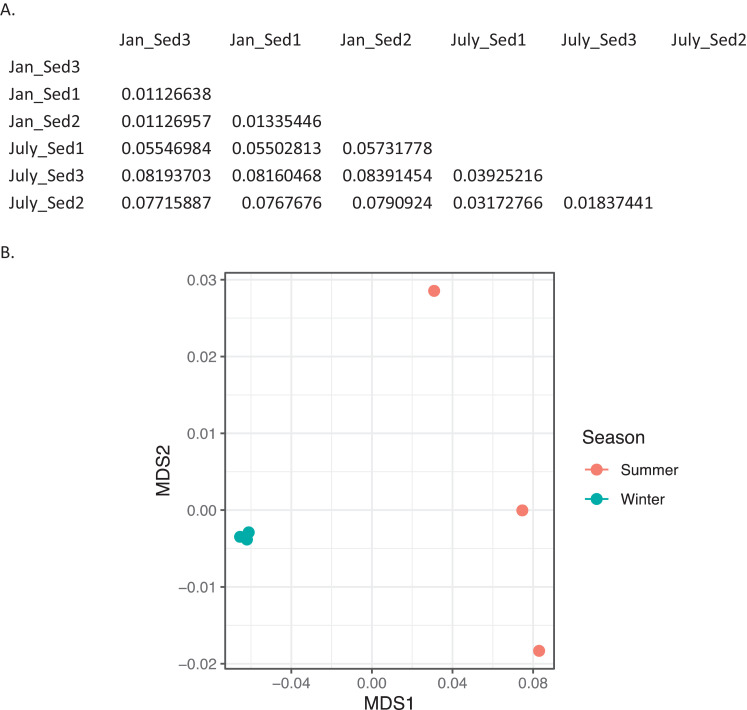
*Bray-Curtis dissimilarity indices for bacteria in sediment.* A) Matrix of dissimilarity indices calculated for genera of bacteria in sediment samples using the Bray-Curtis method. 'Sed' = sediment. B) NMDS plot to visualize the dissimilarity between genera of bacteria in summer (July_Sed1, July_Sed2, and July_Sed3 in orange) and winter (Jan_Sed1, Jan_Sed2, and Jan_Sed3 in blue) sediment collected at EB-90M. (For interpretation of the references to color in this figure legend, the reader is referred to the web version of this article.)

**Fig. 4:**
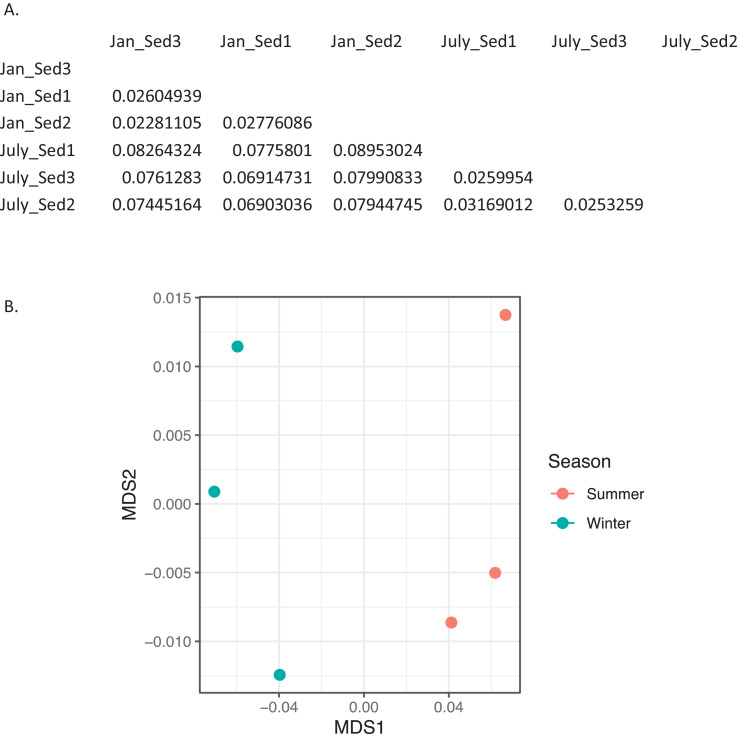
*Bray-Curtis dissimilarity indices for eukaryota in sediment.* A) Matrix of dissimilarity indices calculated for genera of eukaryota in sediment samples using the Bray-Curtis method. 'Sed' = sediment. B) NMDS plot to visualize the dissimilarity between genera of eukaryota in summer (July_Sed1, July_Sed2, and July_Sed3 in orange) and winter sediment (Jan_Sed1, Jan_Sed2, and Jan_Sed3 in blue) collected at EB-90M. (For interpretation of the references to color in this figure legend, the reader is referred to the web version of this article.)

**Fig. 5:**
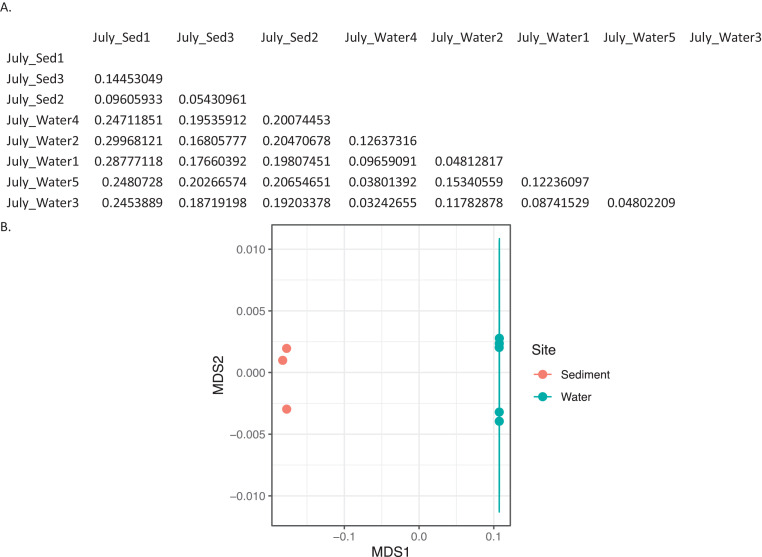
*Bray-Curtis dissimilarity indices for archaea in summer.* A) Matrix of dissimilarity indices calculated for genera of archaea in summer samples using the Bray-Curtis method. 'Sed' = sediment. B) NMDS plot to visualize the dissimilarity between genera of archaea in summer sediment (July_Sed1, July_Sed2, and July_Sed3 in orange) and water (July_Water1, July_Water2, July_Water3, July_Water4, and July_Water5 in blue) collected at EB-90M. The ellipse indicates a clustering of more than 3 samples. (For interpretation of the references to color in this figure legend, the reader is referred to the web version of this article.)

**Fig. 6:**
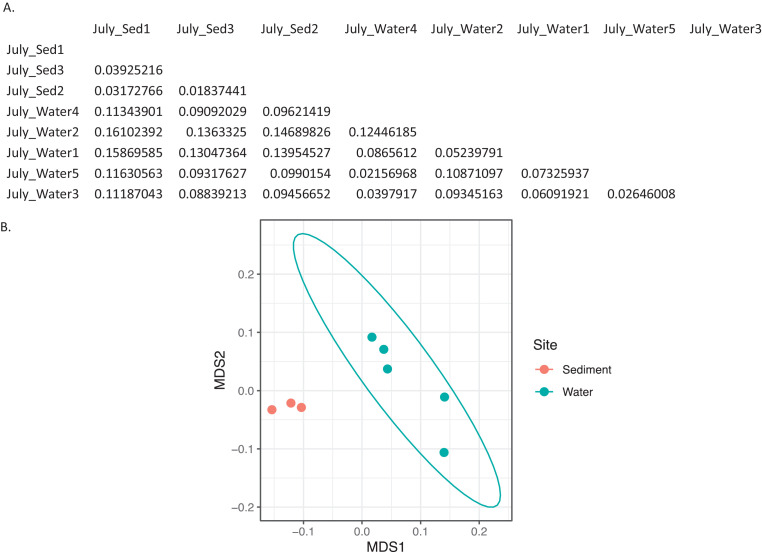
*Bray-Curtis dissimilarity indices for bacteria in summer.* A) Matrix of dissimilarity indices calculated for genera of bacteria in summer samples using the Bray-Curtis method. 'Sed' = sediment. B) NMDS plot to visualize the dissimilarity between genera of bacteria in summer sediment (July_Sed1, July_Sed2, and July_Sed3 in orange) and water (July_Water1, July_Water2, July_Water3, July_Water4, and July_Water5 in blue) collected at EB-90M. The ellipse indicates a clustering of more than 3 samples. (For interpretation of the references to color in this figure legend, the reader is referred to the web version of this article.)

**Fig. 7:**
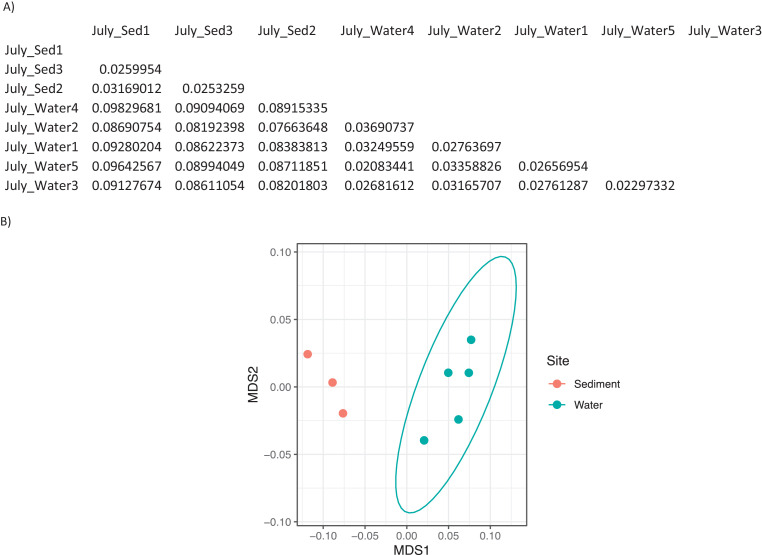
*Bray-Curtis dissimilarity indices for eukaryota in summer.* A) Matrix of dissimilarity indices calculated for genera of eukaryota in summer samples using the Bray-Curtis method. 'Sed' = sediment. B) NMDS plot to visualize the dissimilarity between genera of eukaryota in summer sediment (July_Sed1, July_Sed2, and July_Sed3 in orange) and water (July_Water1, July_Water2, July_Water3, July_Water4, and July_Water5 in blue) collected at EB-90M. The ellipse indicates a clustering of more than 3 samples. (For interpretation of the references to color in this figure legend, the reader is referred to the web version of this article.)

**Fig. 8:**
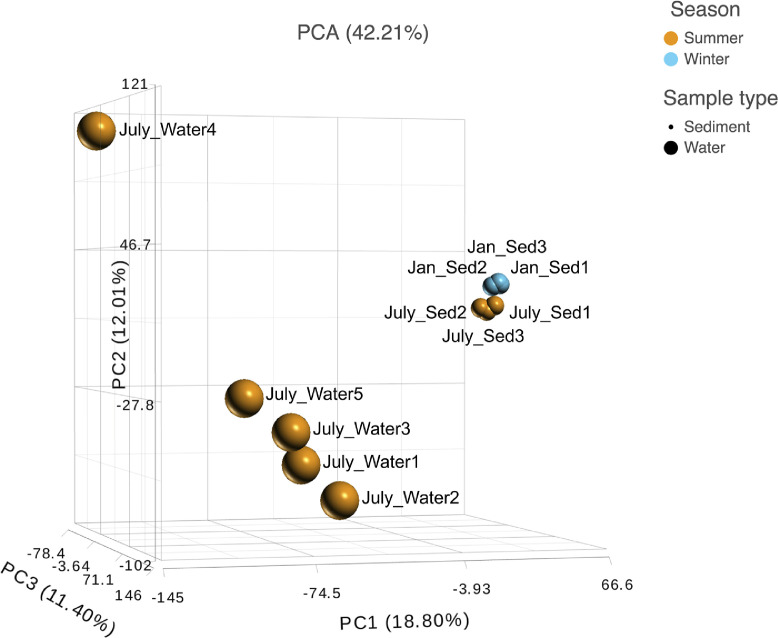
*Taxonomic differences.* PCA plot demonstrating the differences between genera in summer water and sediment as well as summer (orange) and winter (blue) sediment. Plot is based on normalized read counts at the genus level from the taxonomic annotation and quantification of paired-end reads. The sample name notation is based on the month the sample was collected, the sample type (i.e., sediment or water), and individual sample number. ‘Sed’ = sediment. (For interpretation of the references to color in this figure legend, the reader is referred to the web version of this article.)

**Fig. 9:**
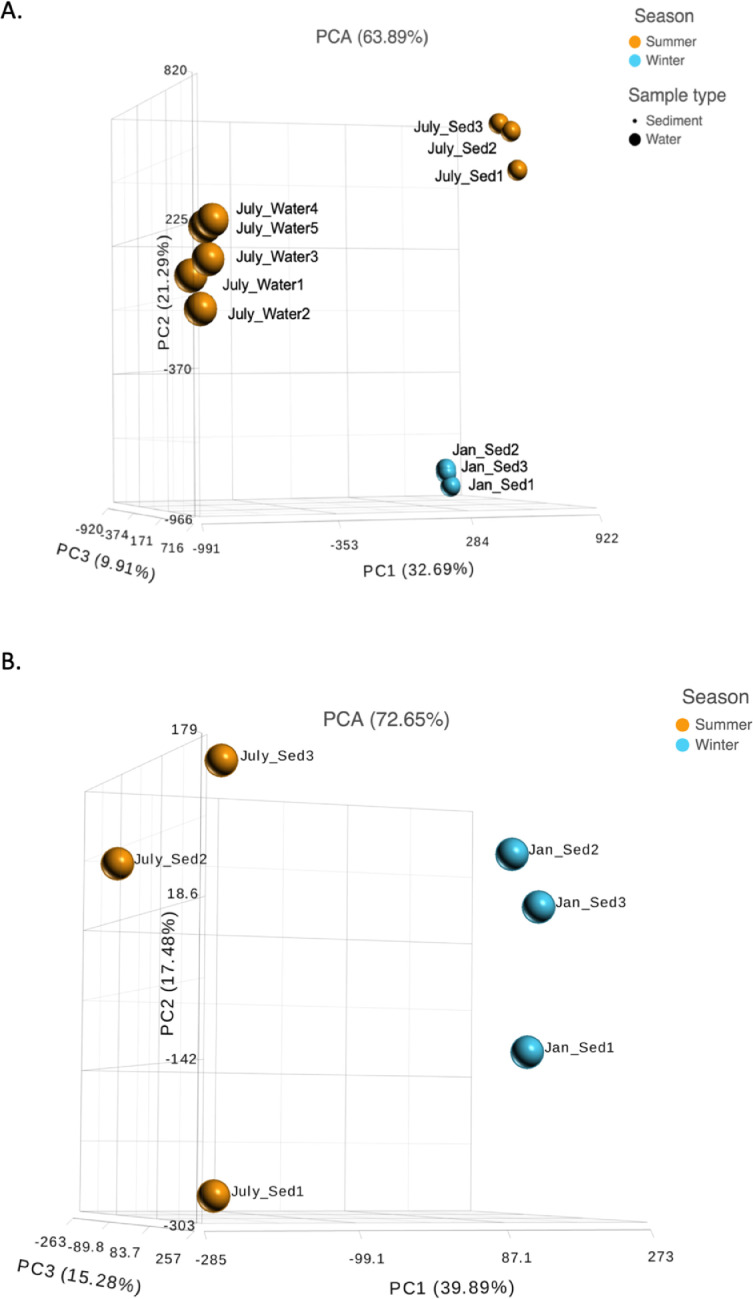
*Differences in DNA and RNA.* PCA plots of A) DNA in water and sediment and B) RNA present in summer and winter sediment based on normalized counts of all functionally annotated genes from the metagenomic assembly, demonstrating differences between sample type. Each gene's normalized read count contributes equally to the PCA. The sample name notation is based on the month the sample was collected, the sample type (i.e., sediment or water), and individual sample number. ‘Sed’ = sediment.

**Fig. 10:**
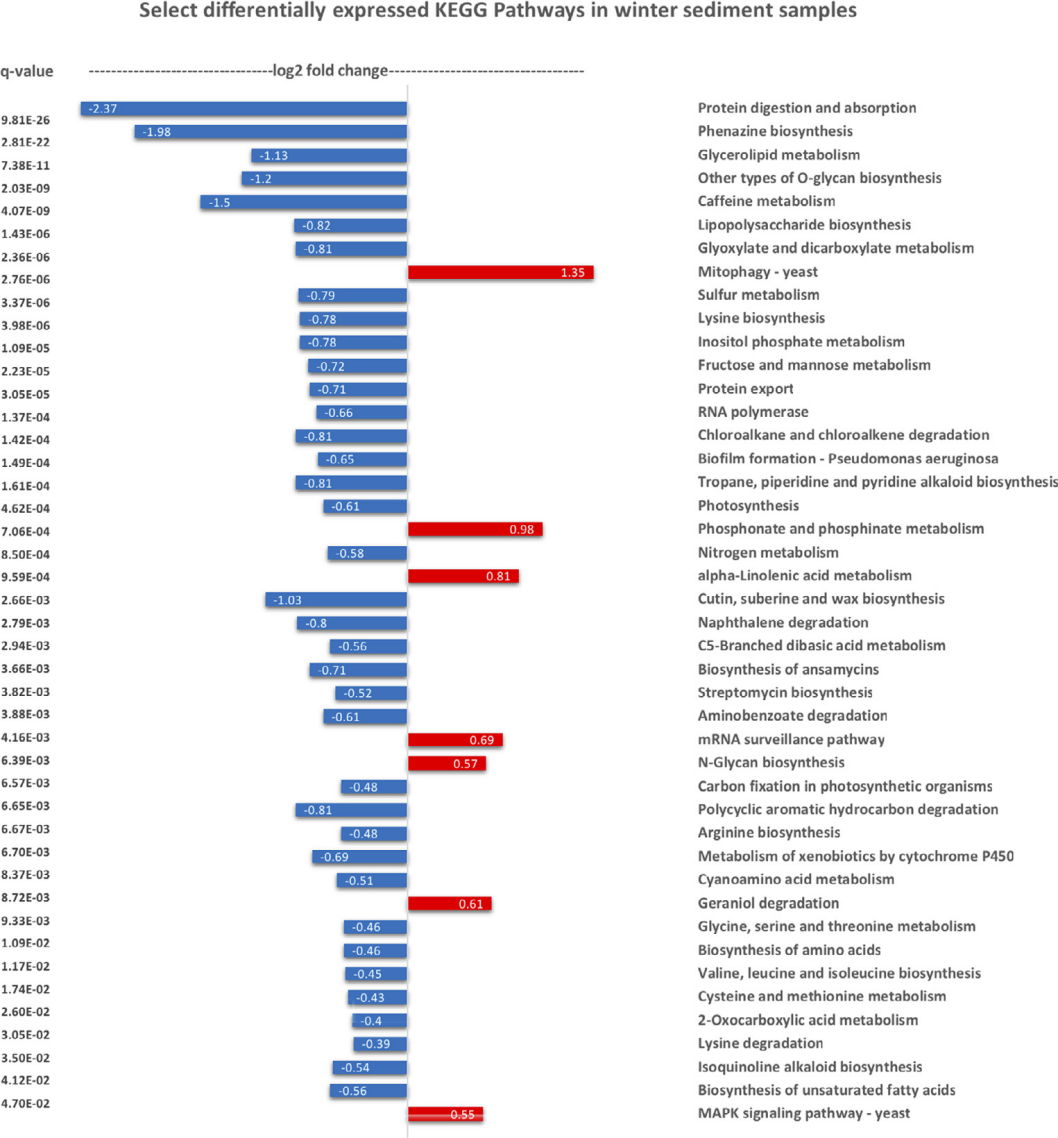
*Significantly differentially xpressed KEGG pathways.* Bar graph of select significantly differentially expressed KEGG pathways in winter versus summer. Differentially expressed pathways were defined based on an unadjusted *p-*value ≤ 0.05 for the interaction term (molecule type-season) in combination with a *q-*winter/summer RNA value ≤ 0.05, respectively. Red and blue represent increased and decreased expression in winter, respectively. (For interpretation of the references to color in this figure legend, the reader is referred to the web version of this article.)

**Fig. 11:**
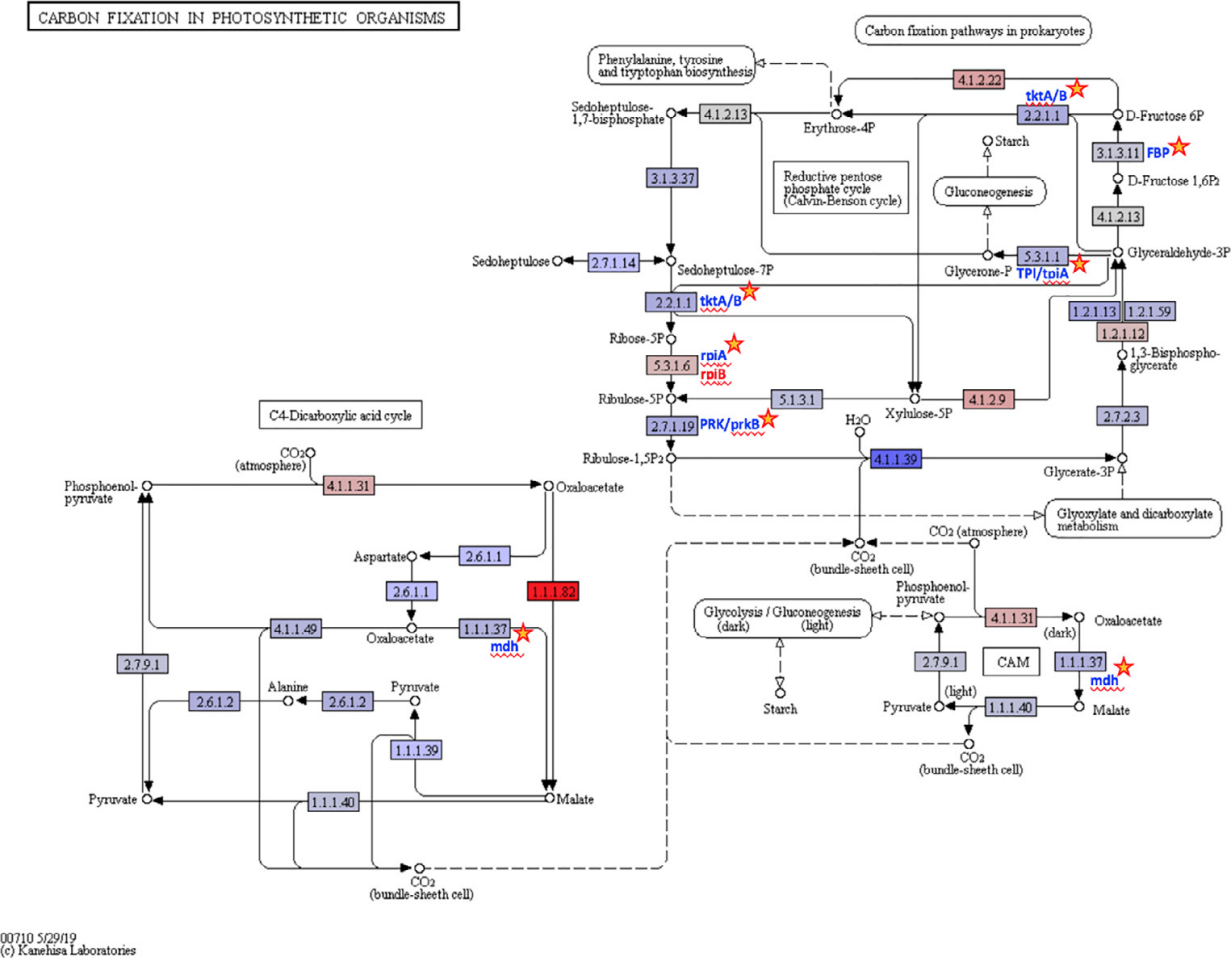
*Carbon fixation in photosynthetic organisms.* Carbon metabolism KEGG reference pathway map (https://www.kegg.jp/pathway/map00710) with color gradation highlighting KEGG genes that change significantly between seasons. Log_2_fold-changes from gene expression analyses were converted to a color gradation using the KEGG Mapper – Color Pathway tool, where blue denotes decreased expression in the winter (RGB color code #6363F7) and red denotes increased expression in the winter (RGB color code #FF000). The Log_2_fold-changes range from −2.33 (blue) to +1.88 (red). Genes with no change in expression are shaded in light gray (RGB color code #D3D3D3) and genes shaded white were undetected in the dataset. Significantly differentially expressed genes are indicated by a star and met the following criteria: *p-*interaction value ≤ 0.05 in combination with a *q-*winter/summer RNA value ≤ 0.05, respectively. (For interpretation of the references to color in this figure legend, the reader is referred to the web version of this article.)

**Fig. 12:**
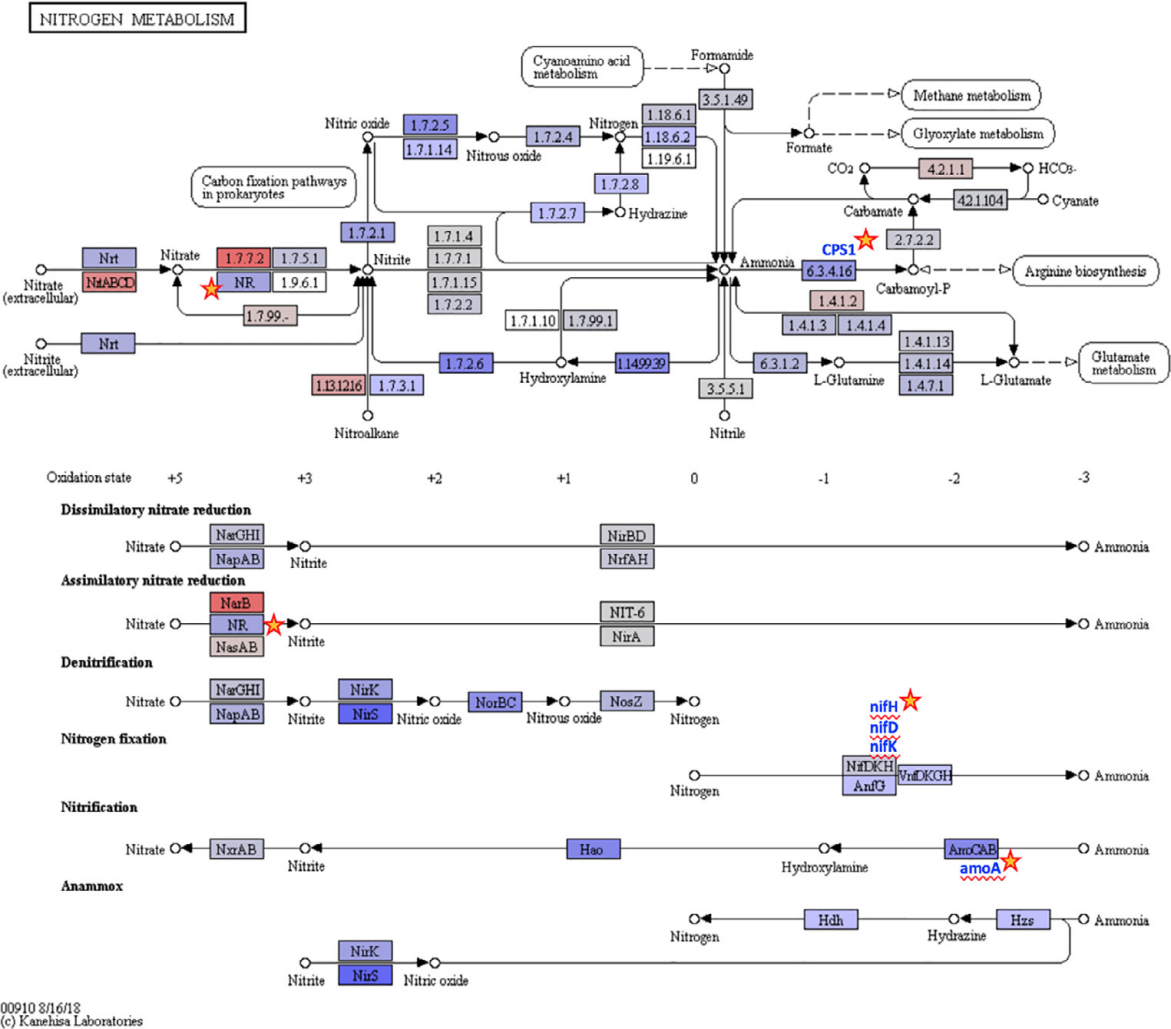
*Nitrogen metabolism gene expression.* Nitrogen metabolism KEGG reference pathway map diagram (https://www.kegg.jp/pathway/map00910) with color gradation highlighting KEGG genes that change significantly between seasons. Log_2_fold-changes from gene expression analyses were converted to a color gradation using the KEGG Mapper – Color Pathway tool, where blue denotes decreased expression in the winter (RGB color code #6363F7) and red denotes increased expression in the winter (RGB color code #FF000). The Log_2_fold-changes range from −3.92 (blue) to +1.91 (red). Genes with no change in expression are shaded in light gray (RGB color code #D3D3D3) and genes shaded white were undetected in the dataset. Significantly differentially expressed genes are indicated by a star and met the following criteria: *p-*interaction value ≤ 0.05 in combination with a *q-*winter/summer RNA value ≤ 0.05, respectively. (For interpretation of the references to color in this figure legend, the reader is referred to the web version of this article.)

**Fig. 13:**
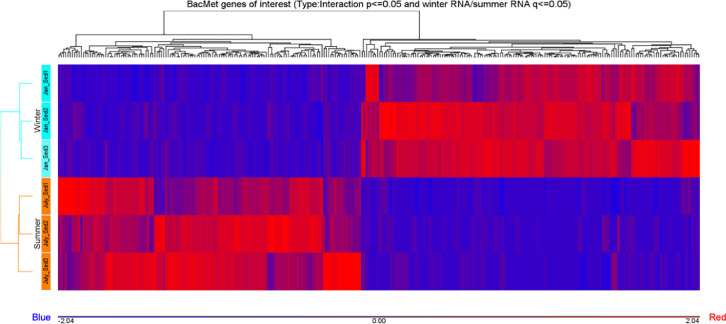
*Metal resistance gene expression.* Hierarchical clustering and heat map of differentially expressed select (288) genes (e.g., *dnaK, copA, copB, copD, pst5, cusA, cusB, mdtA, mdtB, mdtC, actP, mco, ycnJ, corA, csoR*, and *copZ*) from the BacMet database across sediment samples. Increases or decreases in gene expression range from −2.04 (blue) to +2.04 (red). All data met the following criteria: *p-*interaction value ≤ 0.05 in combination with a *q-*winter/summer RNA value ≤ 0.05, respectively. (For interpretation of the references to color in this figure legend, the reader is referred to the web version of this article.)

**Fig. 14:**
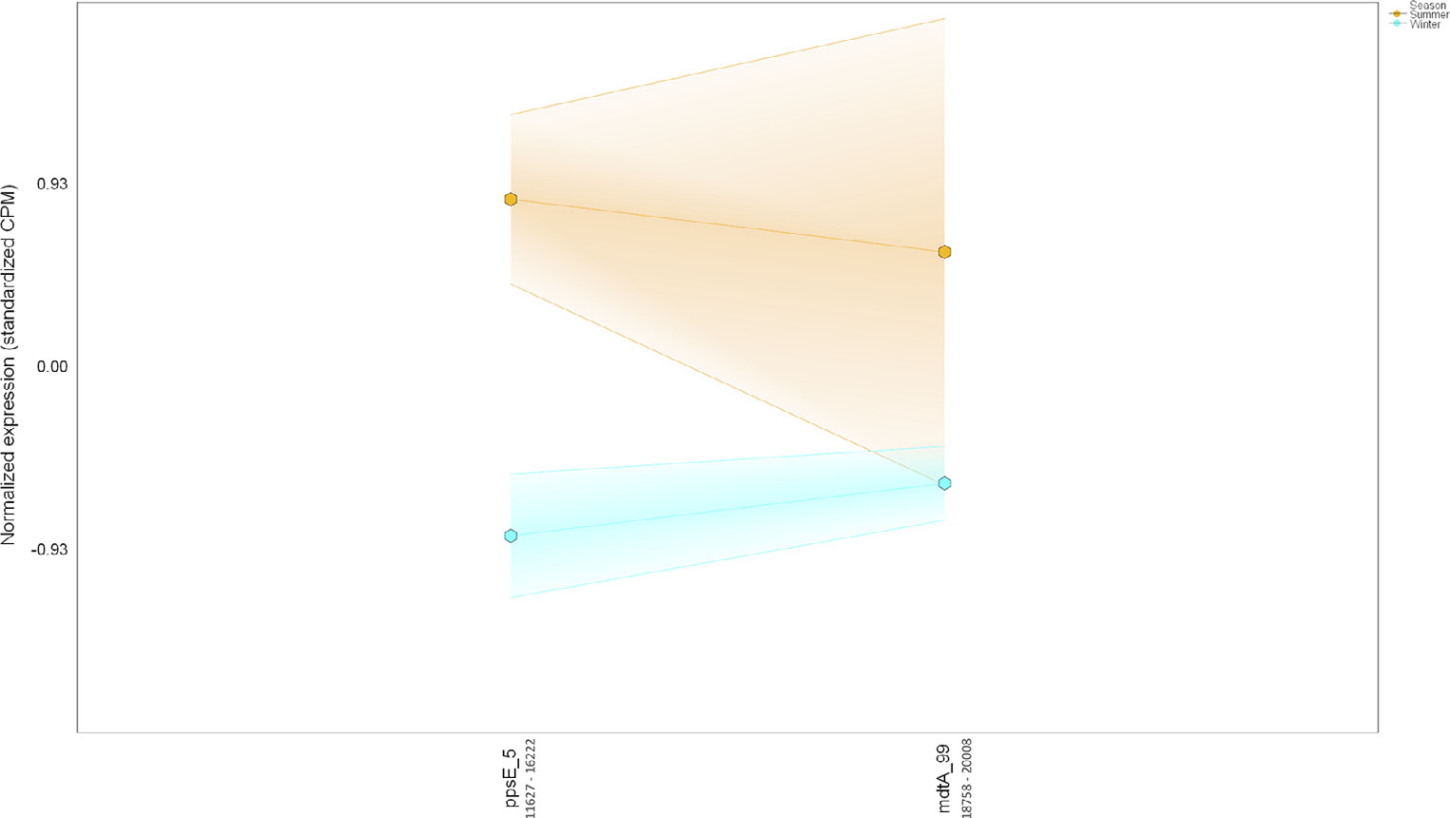
*Colocalization and coexpression of metal resistance and secondary metabolite genes.* Gradient plot demonstrating the differential coexpression of *mdtA*, a metal resistance gene encoding a multidrug resistance protein, with a gene (*ppsE*) annotated to be involved in phthiocerol/phenolphthiocerol polyketide biosynthesis in contig 4698 (20,390 nucleotides long) in summer (orange) and winter (blue). The lines on the y-axis represent the maximum, minimum, and mean of the standardized expression values (i.e., counts per million). All data met the following criteria: *p-*interaction ≤ 0.05 in combination with a *p-*winter/summer RNA ≤ 0.05, respectively. Nucleotide positions in contig are shown below gene IDs. (For interpretation of the references to color in this figure legend, the reader is referred to the web version of this article.)

**Fig. 15:**
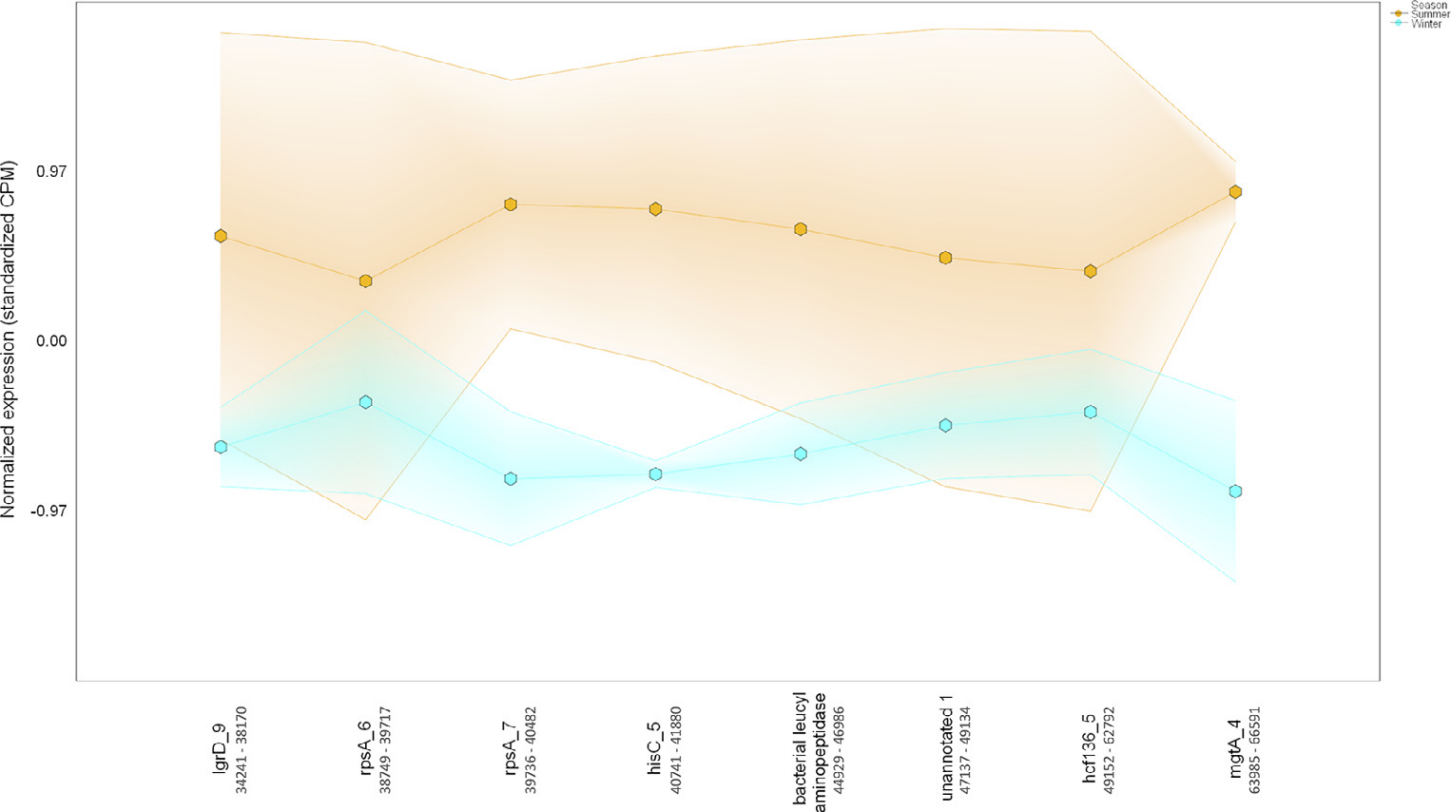
*Colocalization and coexpression of metal resistance and secondary metabolite genes.* Gradient plot demonstrating the differential coexpression of *mgtA*, a metal resistance gene encoding a cation transport ATPase that mediates magnesium influx into the cytosol, with genes (*lgrD*) annotated to be involved in gramicidin biosynthesis in contig 80 (113,676 nucleotides long) in summer (orange) and winter (blue). The lines on the y-axis represent the maximum, minimum, and mean of the standardized expression values (i.e., counts per million). Only *mgtA* met the following criteria: *p-*interaction ≤ 0.05 in combination with a *q-*winter/summer RNA ≤ 0.05, respectively. Nucleotide positions in contig are shown below gene IDs. (For interpretation of the references to color in this figure legend, the reader is referred to the web version of this article.)

**Fig. 16:**
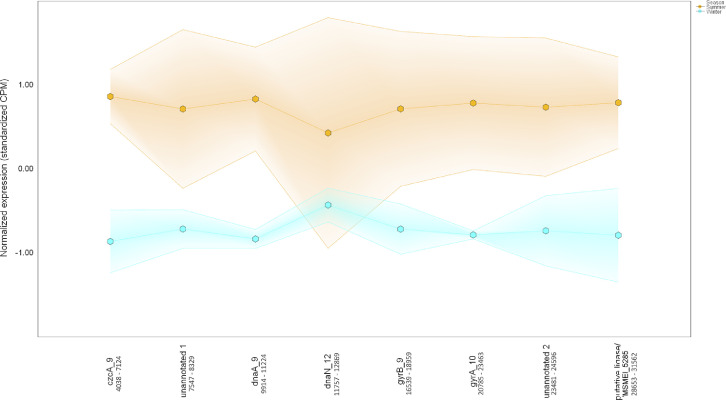
*Colocalization and coexpression of metal resistance and secondary metabolite genes.* Gradient plot demonstrating the differential coexpression of *czcA*, a metal resistance gene encoding a cobalt-zinc-cadmium resistance protein, with a ligase/MSMEI_5285 gene annotated to be involved in the biosynthesis of a polyketide in contig 185 (85,942 nucleotides long) in summer (orange) and winter (blue). The lines on the y-axis represent the maximum, minimum, and mean of the standardized expression values (i.e., counts per million). Only *czcA* met the following criteria: *p-*interaction ≤ 0.05 in combination with a *q-*winter/summer RNA ≤ 0.05, respectively. Nucleotide positions in contig are shown below gene IDs. (For interpretation of the references to color in this figure legend, the reader is referred to the web version of this article.)

**Fig. 17:**
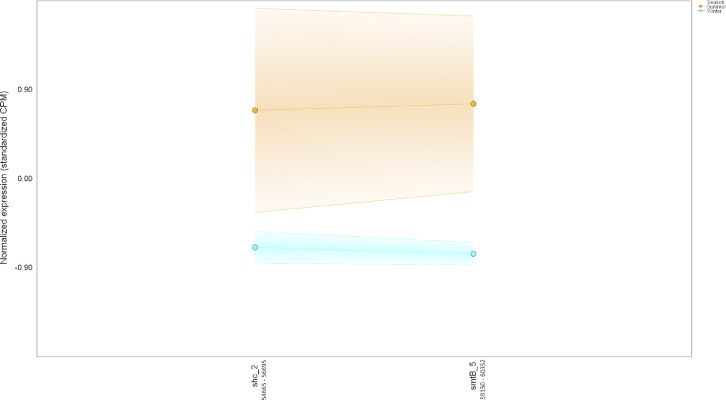
*Colocalization and coexpression of metal resistance and secondary metabolite genes.* Gradient plot demonstrating the differential coexpression of *smtB*, a zinc-resistance gene encoding a repressor protein of the metallothionein gene *smtA*, with a gene annotated to be involved in the biosynthesis of a terpene in contig 214 (80,995 nucleotides long) in summer (orange) and winter (blue). The lines on the y-axis represent the maximum, minimum, and mean of the standardized expression values (i.e., counts per million). Only *SmtB* met the following criteria: *p-*interaction ≤ 0.05 in combination with a *q-*winter/summer RNA ≤ 0.05, respectively. Nucleotide positions in contig are shown below gene IDs. (For interpretation of the references to color in this figure legend, the reader is referred to the web version of this article.)

**Fig. 18:**
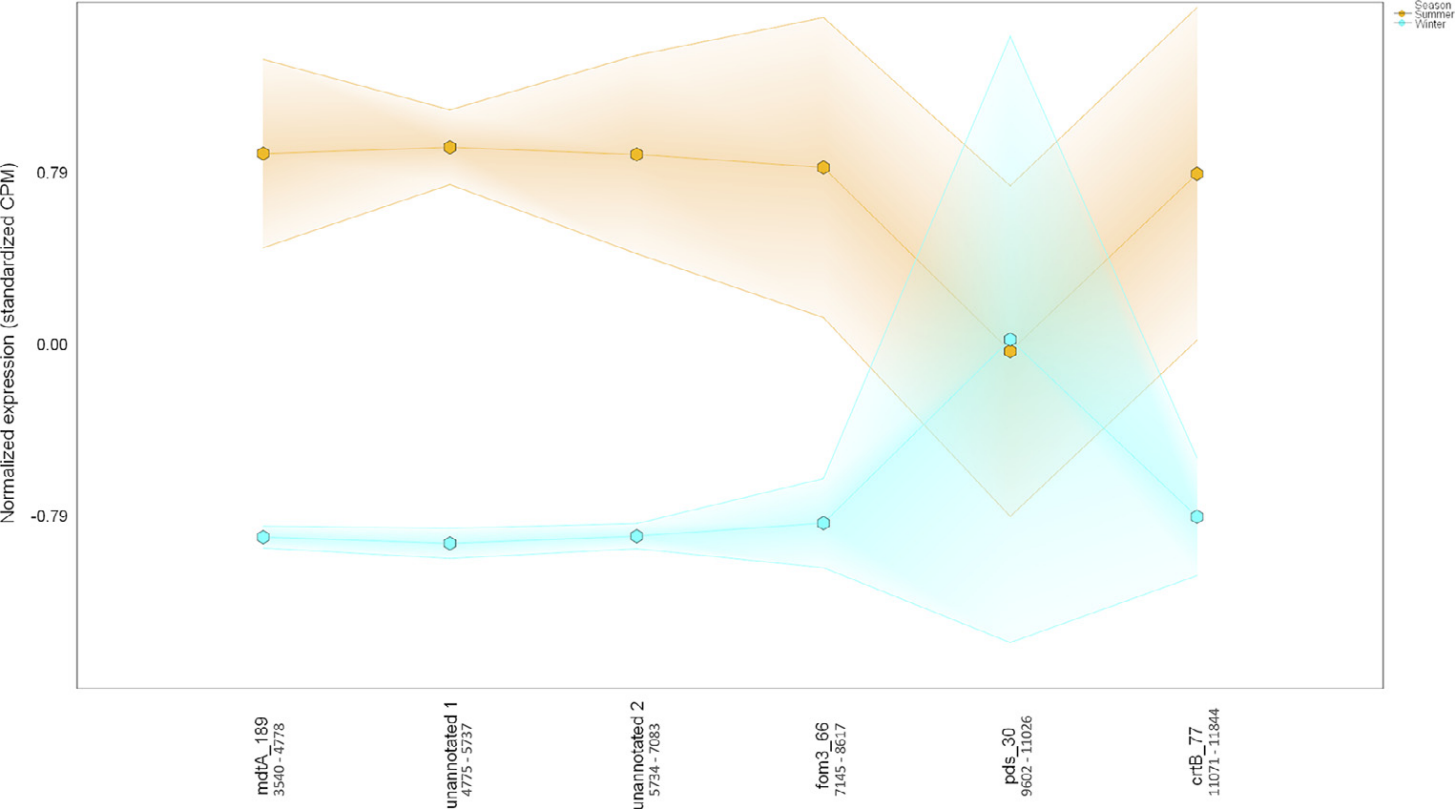
*Colocalization and coexpression of metal resistance and secondary metabolite genes.* Gradient plot demonstrating the differential coexpression of *mdtA*, a metal resistance gene encoding multidrug resistance protein, with genes annotated to be involved in the biosynthesis of a terpene in contig 12,335 (11,958 nucleotides long) in summer (orange) and winter (blue). The lines on the y-axis represent the maximum, minimum, and mean of the standardized expression values (i.e., counts per million). Only *mdtA* met the following criteria: *p-*interaction ≤ 0.05 in combination with a *q-*winter/summer RNA ≤ 0.05, respectively. Nucleotide positions in contig are shown below gene IDs. (For interpretation of the references to color in this figure legend, the reader is referred to the web version of this article.)

Ten water and six sediment samples at Ely Brook (EB-90M) (Fig. 1), Ely Copper Mine Superfund site were collected in July 2017 and January 2018. Shotgun metagenomic sequencing of nucleic acids extracted from water and sediment samples generated ∼31,545,991 reads with an average length of 147 bp and a total length of 1.54 Gb for 11 samples. Samples of the same sample type (i.e., water or sediment) or season (i.e., summer or winter) were treated as biological replicates. Summer water samples were denoted as July_Water1, July_Water2, July_Water3, July_Water4, July_Water5. Summer sediment samples were denoted as July_Sed1, July_Sed2, and July_Sed3. Winter sediment samples were denoted as Jan_Sed1, Jan_Sed2, and Jan_Sed3. All winter water samples (five samples) did not yield viable sequencing data. Of the remaining 11 samples, ∼12 Gb of data (50 M clusters) were produced per sample with an average of 25,181,359 reads per sample over a range of 8,657,966 and 44,323,783 reads for both metagenomic and metatranscriptomic data. Contigs of ≥ 3264 bp (N50) represented 50% of data and the longest contig was 488,568 bp in length. Using Centrifuge [6] to perform read-based taxonomic annotation, 141 distinct phyla were annotated, including candidate phyla (Table 1). Taxonomic differences across season and sample type were observed by NMDS and PCA analyses of normalized count data (i.e., counts per million) between the bacteria, archaea, and fungi in samples as well as molecule types (Figs. 2–8). Differences between molecule type (i.e., DNA or RNA) across sample type and season were assessed by multivariate principal component analyses (PCA) (Fig. 9). Using Prokka-annotated open reading frames [7], Kyoto Encyclopedia of Genes and Genomes (KEGG) reference pathways [8] were annotated and quantified (Table 2). Significantly differentially expressed KEGG pathways and genes in winter versus summer were defined as having winter/summer RNA *p*-values ≤ 0.05 for the interaction of season and molecule type followed by false discovery rate (FDR) corrections [9] (*q*-values) ≤ 0.05 (Figs. 10–12). Secondary metabolite gene clusters (Table 3), metal resistance genes (Table 4), and antibiotic resistance genes were identified (Table 5). Approximately 288 metal resistance genes were differentially expressed between winter and summer seasons (Fig. 13). Furthermore, some of these genes were colocalized and coexpressed with genes involved in secondary metabolism (Table 6; Figs. 14–18).

**Table 1:** *Taxonomic annotation.* List of 141 unique phyla across water and sediment metagenomic samples at EB-90 M. sk, superkingdom; k, kingdom; p, phylum. Incertae sedis represents kingdoms that have not been assigned.

Unique phyla across water and sediment metagenomic samples	
sk_Archaea;k__Archaea incertae sedis;p__Archaea incertae sedis	sk_Bacteria;k__Bacteria incertae sedis;p__Planctomycetes
sk_Archaea;k__Archaea incertae sedis;p__Candidatus Korarchaeota	sk_Bacteria;k__Bacteria incertae sedis;p__Proteobacteria
sk_Archaea;k__Archaea incertae sedis;p__Candidatus Micrarchaeota	sk_Bacteria;k__Bacteria incertae sedis;p__Spirochaetes
sk_Archaea;k__Archaea incertae sedis;p__Candidatus Nanohaloarchaeota	sk_Bacteria;k__Bacteria incertae sedis;p__Synergistetes
sk_Archaea;k__Archaea incertae sedis;p__Candidatus Parvarchaeota	sk_Bacteria;k__Bacteria incertae sedis;p__Tenericutes
sk_Archaea;k__Archaea incertae sedis;p__Crenarchaeota	sk_Bacteria;k__Bacteria incertae sedis;p__Thermodesulfobacteria
sk_Archaea;k__Archaea incertae sedis;p__Euryarchaeota	sk_Bacteria;k__Bacteria incertae sedis;p__Thermotogae
sk_Archaea;k__Archaea incertae sedis;p__Nanoarchaeota	sk_Bacteria;k__Bacteria incertae sedis;p__Verrucomicrobia
sk_Archaea;k__Archaea incertae sedis;p__Thaumarchaeota	sk_Bacteria;k__Bacteria incertae sedis;p__candidate division CPR2
sk_Bacteria;k__Bacteria incertae sedis;p__Acidobacteria	sk_Bacteria;k__Bacteria incertae sedis;p__candidate division CPR3
sk_Bacteria;k__Bacteria incertae sedis;p__Actinobacteria	sk_Bacteria;k__Bacteria incertae sedis;p__candidate division NC10
sk_Bacteria;k__Bacteria incertae sedis;p__Aquificae	sk_Bacteria;k__Bacteria incertae sedis;p__candidate division WPS-2
sk_Bacteria;k__Bacteria incertae sedis;p__Armatimonadetes	sk_Bacteria;k__Bacteria incertae sedis;p__candidate division WWE3
sk_Bacteria;k__Bacteria incertae sedis;p__Bacteria incertae sedis	sk_Eukaryota;k__Eukaryota incertae sedis;p__Apicomplexa
sk_Bacteria;k__Bacteria incertae sedis;p__Bacteroidetes	sk_Eukaryota;k__Eukaryota incertae sedis;p__Bacillariophyta
sk_Bacteria;k__Bacteria incertae sedis;p__Balneolaeota	sk_Eukaryota;k__Eukaryota incertae sedis;p__Bolidophyceae
sk_Bacteria;k__Bacteria incertae sedis;p__Caldiserica	sk_Eukaryota;k__Eukaryota incertae sedis;p__Chromerida
sk_Bacteria;k__Bacteria incertae sedis;p__Calditrichaeota	sk_Eukaryota;k__Eukaryota incertae sedis;p__Colponemidia
sk_Bacteria;k__Bacteria incertae sedis;p__Candidatus Acetothermia	sk_Eukaryota;k__Eukaryota incertae sedis;p__Euglenida
sk_Bacteria;k__Bacteria incertae sedis;p__Candidatus Adlerbacteria	sk_Eukaryota;k__Eukaryota incertae sedis;p__Eukaryota incertae sedis
sk_Bacteria;k__Bacteria incertae sedis;p__Candidatus Amesbacteria	sk_Eukaryota;k__Eukaryota incertae sedis;p__Eustigmatophyceae
sk_Bacteria;k__Bacteria incertae sedis;p__Candidatus Atribacteria	sk_Eukaryota;k__Eukaryota incertae sedis;p__Haplosporidia
sk_Bacteria;k__Bacteria incertae sedis;p__Candidatus Azambacteria	sk_Eukaryota;k__Eukaryota incertae sedis;p__Phaeophyceae
sk_Bacteria;k__Bacteria incertae sedis;p__Candidatus Beckwithbacteria	sk_Eukaryota;k__Eukaryota incertae sedis;p__Picozoa
sk_Bacteria;k__Bacteria incertae sedis;p__Candidatus Berkelbacteria	sk_Eukaryota;k__Eukaryota incertae sedis;p__Pinguiophyceae
sk_Bacteria;k__Bacteria incertae sedis;p__Candidatus Campbellbacteria	sk_Eukaryota;k__Eukaryota incertae sedis;p__Xanthophyceae
sk_Bacteria;k__Bacteria incertae sedis;p__Candidatus Cloacimonetes	sk_Eukaryota;k__Fungi;p__Ascomycota
sk_Bacteria;k__Bacteria incertae sedis;p__Candidatus Collierbacteria	sk_Eukaryota;k__Fungi;p__Basidiomycota
sk_Bacteria;k__Bacteria incertae sedis;p__Candidatus Curtissbacteria	sk_Eukaryota;k__Fungi;p__Blastocladiomycota
sk_Bacteria;k__Bacteria incertae sedis;p__Candidatus Daviesbacteria	sk_Eukaryota;k__Fungi;p__Chytridiomycota
sk_Bacteria;k__Bacteria incertae sedis;p__Candidatus Falkowbacteria	sk_Eukaryota;k__Fungi;p__Cryptomycota
sk_Bacteria;k__Bacteria incertae sedis;p__Candidatus Giovannonibacteria	sk_Eukaryota;k__Fungi;p__Entorrhizomycota
sk_Bacteria;k__Bacteria incertae sedis;p__Candidatus Gottesmanbacteria	sk_Eukaryota;k__Fungi;p__Fungi incertae sedis
sk_Bacteria;k__Bacteria incertae sedis;p__Candidatus Gracilibacteria	sk_Eukaryota;k__Fungi;p__Microsporidia
sk_Bacteria;k__Bacteria incertae sedis;p__Candidatus Jorgensenbacteria	sk_Eukaryota;k__Fungi;p__Mucoromycota
sk_Bacteria;k__Bacteria incertae sedis;p__Candidatus Kaiserbacteria	sk_Eukaryota;k__Fungi;p__Zoopagomycota
sk_Bacteria;k__Bacteria incertae sedis;p__Candidatus Kuenenbacteria	sk_Eukaryota;k__Metazoa;p__Acanthocephala
sk_Bacteria;k__Bacteria incertae sedis;p__Candidatus Levybacteria	sk_Eukaryota;k__Metazoa;p__Annelida
sk_Bacteria;k__Bacteria incertae sedis;p__Candidatus Magasanikbacteria	sk_Eukaryota;k__Metazoa;p__Arthropoda
sk_Bacteria;k__Bacteria incertae sedis;p__Candidatus Melainabacteria	sk_Eukaryota;k__Metazoa;p__Brachiopoda
sk_Bacteria;k__Bacteria incertae sedis;p__Candidatus Moranbacteria	sk_Eukaryota;k__Metazoa;p__Bryozoa
sk_Bacteria;k__Bacteria incertae sedis;p__Candidatus Nomurabacteria	sk_Eukaryota;k__Metazoa;p__Chaetognatha
sk_Bacteria;k__Bacteria incertae sedis;p__Candidatus Omnitrophica	sk_Eukaryota;k__Metazoa;p__Chordata
sk_Bacteria;k__Bacteria incertae sedis;p__Candidatus Pacebacteria	sk_Eukaryota;k__Metazoa;p__Cnidaria
sk_Bacteria;k__Bacteria incertae sedis;p__Candidatus Parcubacteria	sk_Eukaryota;k__Metazoa;p__Ctenophora
sk_Bacteria;k__Bacteria incertae sedis;p__Candidatus Peregrinibacteria	sk_Eukaryota;k__Metazoa;p__Cycliophora
sk_Bacteria;k__Bacteria incertae sedis;p__Candidatus Roizmanbacteria	sk_Eukaryota;k__Metazoa;p__Echinodermata
sk_Bacteria;k__Bacteria incertae sedis;p__Candidatus Saccharibacteria	sk_Eukaryota;k__Metazoa;p__Entoprocta
sk_Bacteria;k__Bacteria incertae sedis;p__Candidatus Shapirobacteria	sk_Eukaryota;k__Metazoa;p__Gastrotricha
sk_Bacteria;k__Bacteria incertae sedis;p__Candidatus Tectomicrobia	sk_Eukaryota;k__Metazoa;p__Gnathostomulida
sk_Bacteria;k__Bacteria incertae sedis;p__Candidatus Uhrbacteria	sk_Eukaryota;k__Metazoa;p__Hemichordata
sk_Bacteria;k__Bacteria incertae sedis;p__Candidatus Woesebacteria	sk_Eukaryota;k__Metazoa;p__Kinorhyncha
sk_Bacteria;k__Bacteria incertae sedis;p__Candidatus Wolfebacteria	sk_Eukaryota;k__Metazoa;p__Metazoa incertae sedis
sk_Bacteria;k__Bacteria incertae sedis;p__Candidatus Yanofskybacteria	sk_Eukaryota;k__Metazoa;p__Mollusca
sk_Bacteria;k__Bacteria incertae sedis;p__Chlamydiae	sk_Eukaryota;k__Metazoa;p__Nematoda
sk_Bacteria;k__Bacteria incertae sedis;p__Chlorobi	sk_Eukaryota;k__Metazoa;p__Nematomorpha
sk_Bacteria;k__Bacteria incertae sedis;p__Chloroflexi	sk_Eukaryota;k__Metazoa;p__Nemertea
sk_Bacteria;k__Bacteria incertae sedis;p__Chrysiogenetes	sk_Eukaryota;k__Metazoa;p__Onychophora
sk_Bacteria;k__Bacteria incertae sedis;p__Coprothermobacterota	sk_Eukaryota;k__Metazoa;p__Placozoa
sk_Bacteria;k__Bacteria incertae sedis;p__Cyanobacteria	sk_Eukaryota;k__Metazoa;p__Platyhelminthes
sk_Bacteria;k__Bacteria incertae sedis;p__Deferribacteres	sk_Eukaryota;k__Metazoa;p__Porifera
sk_Bacteria;k__Bacteria incertae sedis;p__Deinococcus-Thermus	sk_Eukaryota;k__Metazoa;p__Priapulida
sk_Bacteria;k__Bacteria incertae sedis;p__Dictyoglomi	sk_Eukaryota;k__Metazoa;p__Rhombozoa
sk_Bacteria;k__Bacteria incertae sedis;p__Elusimicrobia	sk_Eukaryota;k__Metazoa;p__Rotifera
sk_Bacteria;k__Bacteria incertae sedis;p__Fibrobacteres	sk_Eukaryota;k__Metazoa;p__Tardigrada
sk_Bacteria;k__Bacteria incertae sedis;p__Firmicutes	sk_Eukaryota;k__Metazoa;p__Xenacoelomorpha
sk_Bacteria;k__Bacteria incertae sedis;p__Fusobacteria	sk_Eukaryota;k__Viridiplantae;p__Chlorophyta
sk_Bacteria;k__Bacteria incertae sedis;p__Gemmatimonadetes	sk_Eukaryota;k__Viridiplantae;p__Streptophyta
sk_Bacteria;k__Bacteria incertae sedis;p__Ignavibacteriae	sk_Viroids;k__Viroids incertae sedis;p__Viroids incertae sedis
sk_Bacteria;k__Bacteria incertae sedis;p__Kiritimatiellaeota	sk_Viruses;k__Viruses incertae sedis;p__Viruses incertae sedis
sk_Bacteria;k__Bacteria incertae sedis;p__Nitrospirae

**Table 2:** *BRITE level 1 annotation statistics.* Average percentages of normalized counts that were annotated at BRITE level 1 using the KEGG database.

Average BRITE level 1 Observations Across All Sediment Samples	Percentage, %
09100 Metabolism	0.1726149
09120 Genetic Information Processing	0.036885
09130 Environmental Information Processing	0.0244681
09140 Cellular Processes	0.0197084
09150 Organismal Systems	0.0107033
09160 Human Diseases	0.020198
09180 BRITE Hierarchies	0.1912439
09190 Not Included in Pathway or BRITE	0.0202365
Unassigned	0.5039421

**Table 3:** *antiSMASH annotation.* Summary of the number of genes and gene clusters annotated by antiSMASH 5.0 as well as those that match the Prokka-annotated data.

Total count of contigs	575,933
Total number of contigs annotated by antiSMASH	1589
Total number of contigs not annotated by antiSMASH	574,344
antiSMASH annotated genes	10,579
antiSMASH annotated genes that aligned with PROKKA analyzed data	4977
antiSMASH annotated genes that did not align with PROKKA analyzed data	5602
antiSMASH annotated gene clusters that align with PROKKA analyzed data	1349
antiSMASH annotated gene clusters that did not align with PROKKA analyzed data	240
antiSMASH annotated gene clusters that aligned with PROKKA analyzed data and met the criteria of a sum of at least 100 counts across all samples and 10 counts in three samples	449
Annotated gene clusters that remain after filtering by *p-*interaction value	176
Annotated gene clusters that remain after subsequent filtering by *q-*winter/summer RNA value	65

**Table 4:** *Metal resistance gene annotation.* Statistics on metal resistance genes identified using the BacMet database. A gene identifier (i.e., gene ID) is defined as a gene symbol plus a number, for example, copR_X, where X is a number. The eight missing gene IDs that were not expressed, include *copR_13, corC_121, cusR_32, czcA_647, nikE_38, pstC_144, ruvB_54, Int_122*. Differentially expressed features were defined based on 1) the interaction term *p-*value (Type:Season) of 0.05 or less in combination with 2) the pairwise seasonal comparison of RNA expression ('Winter.rna/Summer.rna') FDR-adjusted *p-*value (*q-*value) of 0.05 or less.

DNA and RNA	296,476
DNA and RNA with gene IDs	161,984
Number of gene symbols found in DNA and RNA	5579
Number of gene symbols found in DNA and RNA in BacMet database	133
Number of gene IDs from DNA and RNA found in BacMet	7021
Number of gene IDs from DNA in BacMet database that are not found in RNA	8 (*copR_13, corC_121, cusR_32, czcA_647, nike_38, pstC_144, ruvB_54, Int_122*)
Number of gene IDs that are differentially expressed	947

**Table 5:** *ARTS annotated contigs.* ARTs (https://arts3.ziemertlab.com) annotated contigs using Actinobacteria and Alphaproteobacteria reference sets. Phylogeny is not applicable (N/A) to this metagenomic dataset. These data are also located on Figshare; DOI: 10.6084/m9.figshare.c.11879226. URL – https://doi.org/10.6084/m9.figshare.c.11879226).

Reference Set: Actinobacteria																			
																		Totals	
Contigs	1–3712	3713–4241	4242–9999	10,000–15,484	15,485–25,000	25,001–35,573	35,574–45,000	45,001–66,477	66,478–85,000	85,001–110,409	110,410–135,000	135,001–169,688	169,689–239,999	240,000–329,999	330,000–439,999	440,000–501,399	501,400–579,964		
Total Genes	162,298	14,102	98,404	65,200	88,082	78,662	59,473	115,925	84,874	101,037	86,501	108,789	188,391	205,523	218,732	111,940	130,993	Total Genes	1,918,926
Core Essential Genes	395	303	387	387	387	383	381	392	377	384	379	382	399	391	370	333	328	Core Essential Genes	6358
Total BGC Hits	136	9	98	81	90	74	70	119	74	95	75	80	134	142	139	92	81	Total BCGC Hits	1589
Known Resistance Models	944	71	595	411	526	420	331	580	406	473	332	474	683	744	742	346	423	Known Resistance Models	8501
Gene Duplication	364	198	354	342	351	344	336	350	334	344	334	345	355	347	324	289	284	Gene Duplication	5595
BGC Proximity	198	0	44	28	20	10	11	12	2	4	1	2	1	0	0	0	1	BGC Proximity	334
Phylogeny/ HGT	N/A	N/A	N/A	N/A	N/A	N/A	N/A	N/A	N/A	N/A	N/A	N/A	N/A	N/A	N/A	N/A	N/A	Phylogeny/ HGT	0
2 or more	128	0	36	25	16	8	9	11	2	4	1	2	1	0	0	0	1	2 or more	244
3 or more	0	0	0	0	0	0	0	0	0	0	0	0	0	0	0	0	0	3 or more	0
Reference Set: Alpha Proteobacteria																			
Nodes	1–3712	3713–4241	4242–9999	10,000–15,484	15,485–25,000	25,001–35,573	35,574–45,000	45,001–66,477	66,478–85,000	85,001–110,409	110,410–135,000	135,001–169,688	169,689–239,999	240,000–329,999	330,000–439,999	440,000–501,399	501,400–579,964		
Total Genes	162,298	14,102	98,404	65,200	88,082	78,662	59,473	115,925	84,874	101,037	86,501	108,789	188,391	205,523	218,732	111,940	130,993	Total Genes	1,918,926
Core Essential Genes	516	359	506	495	506	505	504	517	492	509	488	502	510	504	488	444	444	Core Essential Genes	8289
Total BGC Hits	136	9	98	81	90	74	70	119	74	95	75	80	134	142	139	92	81	Total BCGC Hits	1589
Known Resistance Models	944	71	595	411	526	420	331	580	406	473	332	474	683	744	742	346	423	Known Resistance Models	8501
																			0
Gene Duplication	486	216	470	444	461	459	439	473	441	470	437	449	478	472	449	380	371	Gene Duplication	7395
BGC Proximity	220	1	71	40	22	21	13	23	5	8	5	5	2	3	2	0	1	BGC Proximity	442
Phylogeny/ HGT	N/A	N/A	N/A	N/A	N/A	N/A	N/A	N/A	N/A	N/A	N/A	N/A	N/A	N/A	N/A	N/A	N/A	Phylogeny/ HGT	0
2 or more	138	1	56	34	17	12	10	14	3	6	3	4	1	1	2	0	1	2 or more	303
3 or more	0	0	0	0	0	0	0	0	0	0	0	0	0	0	0	0	0	3 or more	0

**Table 6:** *Colocalized and/or coexpressed genes.* Colocalized and/or coexpressed BacMet genes with BGCs. Differentially expressed features were defined based on 1) the interaction term *p-*value (Type:Season) (*p-*interaction) of 0.05 or less in combination with 2) the pairwise seasonal comparison of RNA expression ('Winter.rna/Summer.rna') FDR-adjusted *p-*value (*q-*value) of 0.05 or less.

	Contig #	Genus	Percent match to genus	Gene ID	Gene	Funtion	*p-*interaction value	*p-*winter RNA/summer RNA value	*q-*winter RNA/summer RNA value	Winter RNA/summer RNA Log_2_-fold change
**Cluster 1**										
Metal resistance	4689			FHBHJPKI_167716	*pitA_11*	L-methionine sulfoximine/L-methionine sulfone acetyltransferase	4.37E-05	0.000733	0.00468	−2.94
Secondary metabolite	4689			FHBHJPKI_167725		Involved in synthesis of homoserine lactone-nonribosomal peptide	1.93078E-11	2.85272E-09	5.90633E-08	−3.500187665
**Cluster 2**										
Metal resistance	80	*Candidatus Solibacter usitatus Ellin6076*	100	FHBHJPKI_12377	*mgtA_4*	Magnesium-transporting ATPase-2C P-type 1	0.00701	0.0182	0.0167	−2.01
Secondary metabolite	80	*Candidatus Solibacter usitatus Ellin6076*	100	FHBHJPKI_12365	*lgrD_9*	Linear gramicidin synthase subunit D	0.841648661	0.004247211	0.021296771	−2.463862034
**Cluster 3**										
Metal resistance	12,335	*Acidobacterium capsulatum ATCC 51,196*	33	FHBHJPKI_283288	*mdtA_189*	Multidrug resistance protein MdtA	3.68E-10	1.23E-16	8.92E-15	−4.71
Secondary metabolite	12,335	*Acidobacterium capsulatum ATCC 51,196*	33	FHBHJPKI_283295	*crtB_77*	All-trans-phytoene synthase	0.15602993	0.006446359	0.030262887	−2.056268682
**Cluster 4**										
Metal resistance	214	*Ralstonia solanacearum CMR15*	22	FHBHJPKI_24632	*smtB_5*	Succinyl-CoA-l-malate CoA-transferase beta subunit	0.042	0.000392	0.0027	−4.18
Secondary metabolite	214	*Ralstonia solanacearum CMR15*	22	FHBHJPKI_24627	*shc_2*	Squalene–hopene cyclase	0.067593728	0.000537894	0.003591324	−2.489137791
**Cluster 5**										
Metal resistance	185	*Candidatus Koribacter versatilis Ellin345*	100	FHBHJPKI_22308	*czcA_9*	Cobalt-zinc-cadmium resistance protein CzcA	0.0321	0.0017	0.00968	−2.31
Secondary metabolite	185	*Candidatus Koribacter versatilis Ellin345*	100	FHBHJPKI_22329		Putative ligase/MSMEI_5285	0.270379299	0.031944273	0.111550994	−1.879407266
**Cluster 6**										
Metal resistance	4698			FHBHJPKI_167937	*mdtA_99*	Multidrug resistance protein MdtA	0.0292	0.0299	0.106	−3.16
Secondary metabolite	4698			FHBHJPKI_167934	*ppsE_5*	Involved in synthesis of Phthiocerol/phenolphthiocerol polyketide	0.01973596	0.001581153	0.009156685	−1.99280005

## 3 Experimental Design, Materials and Methods

### 3.1 Sample collection

On July 28th, 2017 and January 14th, 2018, Ely Brook (43°55′9″ N, 72°17′11″ W), 90 m upstream from the mouth of the brook (EB-90M), was sampled along with unsaturated sediment (10 cm deep). The physicochemical properties, nucleic acid extraction, library preparation, and metatranscriptomic and metatranscriptomic sequencing, taxonomic annotation of raw reads, metagenomic assembly, and functional annotations of these samples were reported by Giddings et al. [1].

### 3.2 Statistical comparison of microbial community, DNA, and RNA

EB-90M samples of the same sample type or season were treated as biological replicates. Subsets (i.e., season or sample type) of data were compared to each other in statistical analyses. Beta diversity was evaluated via Bray-Curtis measure of dissimilarity [10] using default parameters in R in the vegan library [11]. Prior to analysis, data were log_10_(*x* + 1) transformed and the resulting dissimilarity indices were used to generate NMDS in R using the metaMDS functions in vegan and ggplot2 library [11, 12]. Multivariate PCAs were performed in Partek Flow software v8.0 to assess sample group variation based on genera using normalized read counts from read-based taxonomic annotations and quantification. Feature counts (e.g., taxon) were standardized prior to the PCA so that the contribution of each feature did not depend on its variance. PCA plots were generated for DNA and RNA using 1) normalized read counts (i.e., fractions for relative abundance) from the metagenomic assembly and 2) normalized read counts from the metatranscriptome, respectively. Heat maps and hierarchal clusters were generated in Partek Flow v8.0 using the following, respectively: 1) normalized counts of taxa from the metagenome and predicted open reading frames (ORFs) across samples and 2) the Euclidean dissimilarity index and average linkage method to cluster similar expression patterns and taxon abundances. The normalized data were standardized to a mean of zero and a standard deviation of 1 prior to hierarchal clustering.

### 3.3 Differential expression and visualization of KEGG pathways

Differentially expressed KEGG pathways were represented by color gradation maps (Figs. S14–S15). Log_2_fold-changes from gene expression analysis results were converted to a color gradation using KEGG Mapper – Color Pathway tool (https://www.genome.jp/kegg/tool/map_pathway3.html), where blue denotes decreased expression in the winter (RGB color code #6363F7) and red denotes increased expression in the winter (RGB color code #FF000). Genes with no change in expression are shaded in light gray (RGB color code #D3D3D3). Genes shaded in white indicates that the gene was undetected in the dataset. The numbers in boxes refer to enzyme nomenclature from the KEGG database. Expression data (i.e., normalized counts) for sediment were fit to a linear model, assuming a negative binomial distribution, that included season (i.e., winter versus summer), molecule type (i.e., RNA versus DNA), as well as the interaction of season and molecule type (*p*-interaction). Pairwise comparison tests of season were performed within and between each data type and *p-*values were FDR-corrected [9]. Significant differentially expressed genes met the following criteria: a molecule type-season interaction term *p*-value of 0.05 or less in combination with an FDR-adjusted *p*-value (*q*-value) of 0.05 or less for the pairwise comparison of winter RNA versus summer RNA. Significant data were indicated by an orange star; however, the overall expression of a node may include other genes.

### 3.4 Analysis of genes involved in natural product biosynthesis, metal resistance, and antibiotic resistance

Contigs were mined for secondary metabolite biosynthetic gene clusters (BGCs) in the bacterial and fungal antiSMASH 5.0 [4] database using default parameters. The BacMet database was used to mine DNA and RNA for experimentally validated metal resistance genes [3]. After filtering annotated-BGCs and BacMet genes that had ≥ 100 raw counts in each sample and at least 10 counts in three or more samples, relative BGC and BacMet gene expression was assessed by comparing the counts of Prokka-annotated transcripts to those of DNA using the criteria described by Giddings et al. [1]. Gradient plots were generated in Partek Flow v8.0 for differentially expressed BGCs and those co-expressed with metal resistance genes. Contigs were also mined for antibiotic resistance genes that were within close proximity or colocalized with BGCs using the Antibiotic Resistant Target Seeker (ARTS) version 2 [5] using default parameters. Duplication and BGC proximity, resistance model screens, and genomes that mapped to the following reference phyla were selected: Actinobacteria and Alphaproteobacteria.

## Declaration of Competing Interest

The authors declare that they have no known competing financial interests or personal relationships which have, or could be perceived to have, influenced the work reported in this article.

## Appendix Supplementary materials

Image, application 1

## References

[1] Giddings L.-.A., Chlipala G., Kunstman K., Green S., Morillo K., Bhave K., Peterson H., Driscoll H., Maienschein-Cline M. (2020). Characterization of an acid rock drainage microbiome and transcriptome at the Ely Copper Mine Superfund site. PLoS ONE.

[2] McCarthy D.J., Chen Y., Smyth G.K. (2012). Differential expression analysis of multifactor RNA-Seq experiments with respect to biological variation. Nucl. Acids Res..

[3] Pal C., Bengtsson-Palme J., Rensing C., Kristiansson E., Larsson D.G.J. (2013). BacMet: antibacterial biocide and metal resistance genes database. Nucl. Acids Res..

[4] Blin K., Shaw S., Steinke K., Villebro R., Ziemert N., Lee S.Y., Medema M.H., Weber T. (2019). AntiSMASH 5.0: updates to the secondary metabolite genome mining pipeline. Nucl. Acids Res..

[5] Alanjary M., Kronmiller B., Adamek M., Blin K., Weber T., Huson D., Philmus B., Ziemert N. (2017). The Antibiotic resistant target seeker (ARTS), an exploration engine for antibiotic cluster prioritization and novel drug target discovery. Nucl. Acids Res..

[6] Kim D., Song L., Breitwieser F.P., Salzberg S.L. (2016). Centrifuge: rapid and sensitive classification of metagenomic sequences. Genome Res..

[7] Seemann T. (2014). Prokka: rapid prokaryotic genome annotation. Bioinformatics.

[8] Kanehisa M., Sato Y., Kawashima M., Furumichi M., Tanabe M. (2016). KEGG as a reference resource for gene and protein annotation. Nucl. Acids Res..

[9] Benjamini Y., Hochberg Y. (1995). Controlling the false discovery rate: a practical and powerful approach to multiple testing. J. R. Stat. Soc. Ser. B..

[10] Bray J.R., Curtis J.T. (1957). An ordination of the upland forest communities of Southern Wisconsin. Ecol. Monogr..

[11] Oksanen J. (2018). Vegan: An introduction to ordination.

[12] Wickham H. (2009). Ggplot2.

